# A Scenario-Based Investigation of Surrogate Decision Making for Older Adults

**DOI:** 10.1093/geroni/igaa057.2469

**Published:** 2020-12-16

**Authors:** Yuxin Zhao, Benjamin Katz, Pamela Teaster

**Affiliations:** 1 Booz Allen Hamilton, Bethesda, Maryland, United States; 2 Virginia Tech, Blacksburg, Virginia, United States; 3 Virginia Tech, BLACKSBURG, Virginia, United States

## Abstract

Surrogate decisions involve complex, challenging choices; surrogate decision-makers make treatment decisions for approximately 40% of hospitalized adults and 70% of older adults, and up to 95% of critically ill adults of any age. The purpose of our study was to understand how people make decisions for others and how surrogate decision making is linked to people’s cognition, self-efficacy, and demographics, especially differences in acute (e.g., health and medical care, financial management, and end of life) versus general scenarios (spending time with family, contacting an insurance company on behalf of a family member). Participants were recruited through Amazon’s Mechanical Turk. We collected data from 290 adult participants aged 18 years or older. On average, people reported a higher level of confidence in general versus acute scenario. The differences of confidence in scenario-based surrogate decision-making links to decision-makers’ cognition, self-efficacy, the experience of decision-making, the experience of caregiving, and demographic factors.

